# Incretin-Based Therapy and Thyroid Cancer Risk: A Systematic Review and Meta-Analysis of Randomized Controlled Trials

**DOI:** 10.1016/j.aed.2026.03.003

**Published:** 2026-03-12

**Authors:** Naseem Eisa, Omar Barood

**Affiliations:** 1 Community Health Partners, Fresno, California; 2 Faculty of Medicine, Damascus University, Damascus, Syria

**Keywords:** GLP-1 receptor agonist, tirzepatide, thyroid cancer, meta-analysis, randomized controlled trial, drug safety

## Abstract

**Background:**

The association between incretin-based therapies, including glucagon-like peptide-1 receptor agonists and dual glucose-dependent insulinotropic polypeptide/glucagon-like peptide-1 receptor agonists, and thyroid cancer risk remains controversial. Conflicting observational findings highlight the need for evidence derived exclusively from randomized controlled trials (RCTs).

**Objectives:**

To evaluate the association between incretin-based therapies and thyroid cancer risk using RCT data.

**Methods:**

We conducted a systematic review and meta-analysis following Preferred Reporting Items for Systematic Reviews and Meta-Analyses guidelines. PubMed, EMBASE, and ClinicalTrials.gov were searched through January 4, 2026, for RCTs of approved incretin-based therapies with ≥26 weeks of follow-up that reported thyroid cancer events. Data extraction was performed independently by 2 reviewers. Risk of bias was assessed using the Cochrane Risk of Bias 2 tool. Pooled odds ratios and 95% CIs were calculated using a random-effects model. Certainty of evidence was assessed using the Grading of Recommendations Assessment, Development and Evaluation framework.

**Results:**

Fifteen RCTs enrolling 84 237 participants were included. Across all trials, 28 thyroid cancer events occurred in the incretin-based therapy groups and 15 in the control groups. Meta-analysis showed no statistically significant association between incretin-based therapy use and thyroid cancer risk (odds ratio 1.52, 95% CI 0.86–2.68; I^2^ = 0.0%). Findings were consistent across prespecified subgroup and sensitivity analyses. Certainty of evidence was rated very low due to serious imprecision related to rare events.

**Conclusions:**

RCT evidence does not demonstrate a statistically significant increase in thyroid cancer risk with incretin-based therapies. However, limited follow-up and imprecision prevent exclusion of a clinically relevant increase, supporting the need for long-term surveillance.

## Graphical abstract

**Figure:**
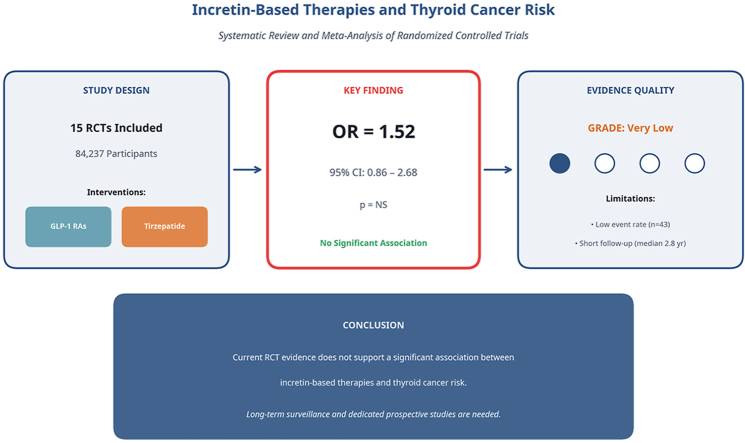

**Highlights**

• This meta-analysis of 15 large-scale randomized controlled trials (*n* = 84 237) provides the most up-to-date evidence on incretin-based therapies and thyroid cancer risk
• Use of glucagon-like peptide-1 receptor agonists or dual glucose-dependent insulinotropic polypeptide/glucagon-like peptide-1 agonists was not associated with a statistically significant increase in thyroid cancer risk (odds ratio 1.52, 95% CI 0.86-2.68)
• The analysis showed no significant heterogeneity between studies (I^2^ = 0%), suggesting consistent findings across trials
• The overall certainty of evidence was rated as "Very Low" by Grading of Recommendations Assessment, Development and Evaluation, primarily due to imprecision from the low number of events (43 total)

**Clinical Relevance**

Current evidence from randomized controlled trials does not support a causal link between the use of incretin-based therapies and an increased risk of thyroid cancer. These findings can help clinicians in counseling patients about the potential risks and benefits of these widely used medications for diabetes and obesity. The very low certainty of evidence underscores the need for continued long-term surveillance and dedicated prospective studies to definitively rule out any potential risk.

## Introduction

The global burden of type 2 diabetes and obesity has reached epidemic proportions, creating an urgent need for effective and safe therapeutic options^1^. Incretin-based therapies, which include glucagon-like peptide-1 receptor agonists (GLP-1RAs) and the newer dual glucose-dependent insulinotropic polypeptide (GIP)/GLP-1RA agents, have emerged as a cornerstone of management for these conditions^2^. Their robust glycemic control, substantial weight loss effects, and proven cardiovascular benefits have led to their widespread adoption in clinical practice worldwide^3^^,^^4^.

Despite these benefits, a persistent safety concern has shadowed the drug class: a potential association with thyroid cancer. This concern originates from preclinical studies in rodents, where long-term administration of GLP-1RAs led to a dose-dependent increase in thyroid C-cell tumors, including medullary thyroid carcinoma (MTC) ^3^. The biological mechanism is thought to involve the stimulation of GLP-1 receptors expressed on rodent thyroid C-cells, leading to cellular proliferation. While human thyroid C-cells express GLP-1 receptors at much lower levels than rodents, the theoretical risk prompted the U.S. Food and Drug Administration to issue a boxed warning for all drugs in this class, advising against their use in patients with a personal or family history of MTC or Multiple Endocrine Neoplasia syndrome type 2 (MEN 2).

The clinical relevance of this finding in humans remains highly controversial. The body of evidence is a mix of conflicting results. Several large-scale observational studies, leveraging real-world data from millions of patients, have suggested a small but statistically significant increase in the risk of thyroid cancer, particularly papillary thyroid cancer, among users of GLP-1RAs^5^^,^^6^. Conversely, other observational studies and initial meta-analyses of randomized controlled trials (RCTs) have found no such association^7^^,^^8^. These discrepancies may be attributable to differences in study design, patient populations, follow-up duration, and the specific drugs analyzed.

Most recently, the approval and widespread use of tirzepatide, a dual GIP/GLP-1RA with even greater efficacy, has added another layer of complexity. As this is a newer agent, data on its long-term safety profile, particularly regarding thyroid cancer, are still emerging^4^. Given the conflicting evidence and the millions of patients currently using these medications, there is a critical need for a comprehensive and up-to-date synthesis of the highest-quality evidence available.

Therefore, the objective of this systematic review and meta-analysis was to address the following PICO question: In patients with or without type 2 diabetes or obesity (P), is treatment with incretin-based therapies (I) compared to placebo or active comparator (C) associated with an increased risk of thyroid cancer (O)? We aimed to provide the most robust and current estimate of this risk by pooling all available data from published RCTs.

## Methods

### Protocol and Registration

This systematic review and meta-analysis was conducted and reported in accordance with the Preferred Reporting Items for Systematic Reviews and Meta-Analyses (PRISMA) 2020 statement^9^. The study protocol was prospectively registered in the International Prospective Register of Systematic Reviews (PROSPERO), registration number CRD420261279687.

### Eligibility Criteria

We included studies that met the following criteria: 1. Study Design: RCTs. 2. Population: Adults (age ≥18 years) with or without type 2 diabetes or obesity. 3. Intervention: Any approved GLP-1RA (liraglutide, semaglutide, dulaglutide, exenatide, lixisenatide, and albiglutide) or dual GIP/GLP-1RA (tirzepatide) administered for at least 26 weeks. 4. Comparator: Placebo or any active comparator (eg, insulin, sodium-glucose cotransporter-2 inhibitors, and DPP-4 inhibitors). 5. Outcome: Reported incidence of at least 1 event of thyroid cancer (including papillary, follicular, medullary, or anaplastic subtypes).

### Information Sources and Search Strategy

We conducted a comprehensive search of PubMed, EMBASE, and ClinicalTrials.gov from their inception to January 4, 2026. The search strategy combined MeSH terms and keywords for incretin-based therapies and thyroid neoplasms, tailored for each database. The full search strategy for PubMed is provided in Supplementary Table S1. We also manually searched the reference lists of included studies and relevant prior systematic reviews^7^^,^^8^^,^^10^ to identify any additional eligible trials.

### Study Selection Process

Two reviewers (A.I. and M.A.) independently screened titles and abstracts of all retrieved records. Full-text articles of potentially eligible studies were then retrieved and assessed for final inclusion. Any disagreements between the reviewers were resolved by consensus or by consulting a third reviewer (S.R.). A list of studies excluded at the full-text stage, with reasons for exclusion, is provided in Supplementary Table S2.

### Data Extraction Process

From each included study, the same 2 reviewers independently extracted the following data using a standardized data extraction form: Study Characteristics: First author, publication year, trial name/acronym, study duration, and sample size. Participant Characteristics: Baseline age, sex, body mass index, and primary diagnosis (type 2 diabetes or obesity). Intervention and Comparator Details: Specific drug, dose, and comparator agent. Outcome Data: The number of thyroid cancer events and the total number of participants in both the intervention and control arms. Data were cross-checked for accuracy, with discrepancies resolved by consensus.

### Risk of Bias Assessment

The methodological quality of each included RCT was independently assessed by 2 reviewers using the Cochrane Risk of Bias 2 (RoB 2) tool^7^. This tool evaluates 5 domains: 1. Bias arising from the randomization process. 2. Bias due to deviations from intended interventions. 3. Bias due to missing outcome data. 4. Bias in measurement of the outcome. 5. Bias in selection of the reported result. Each domain was judged as “Low risk,” “Some concerns,” or “High risk.” The overall risk of bias for each study was determined by the highest risk level in any single domain. A detailed breakdown of the RoB 2 assessment for each study is provided in Supplementary Table S3.

### Data Synthesis and Statistical Analysis

All statistical analyses were performed using R (version 4.3.1) with the meta package. The primary outcome was the incidence of thyroid cancer. We calculated the odds ratio (OR) and 95% CI for each study. A pooled OR was calculated using the Mantel-Haenszel method with a random-effects model, which accounts for potential heterogeneity between studies. A *P* value <0.05 was considered statistically significant.

Heterogeneity was quantified using the I^2^ statistic, with values of <25%, 25% to 75%, and >75% considered low, moderate, and high heterogeneity, respectively.

Subgroup Analysis: We conducted a prespecified subgroup analysis to compare the risk between GLP-1RAs and the dual GIP/GLP-1RA tirzepatide. A test for subgroup differences (interaction test) was performed to assess whether the effect size differed significantly between the 2 drug classes.

Sensitivity Analysis: To assess the robustness of our findings, we performed a prespecified sensitivity analysis restricted to the large, long-duration cardiovascular outcome trials (CVOTs)^11^, ^12^, ^13^, ^14^, ^15^, ^16^, ^17^, ^18^, which are generally considered to be of the highest methodological quality.

**Fig. 1:**
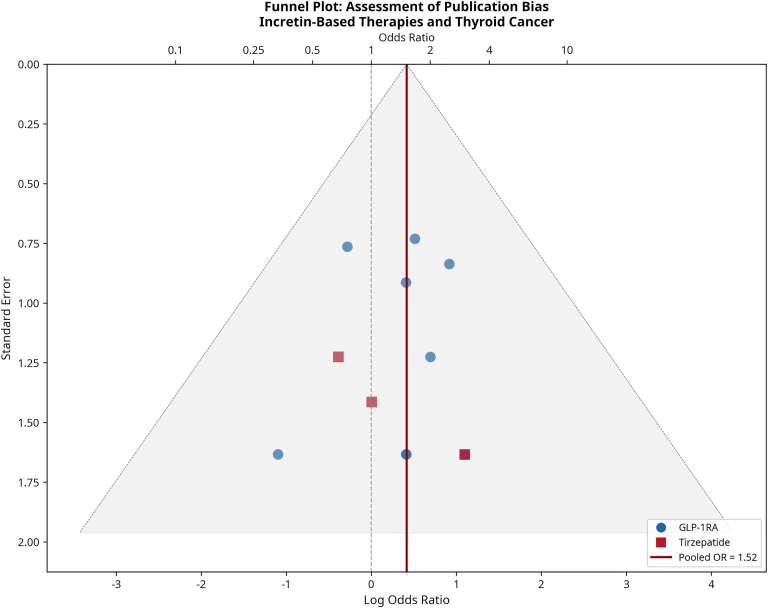
Funnel plot for publication bias assessment. The plot shows the log odds ratio for each study against its standard error. The symmetrical distribution of studies around the pooled estimate suggests no significant publication bias, which was confirmed by Egger’s test (*P* = 0.62). *OR* = odds ratio; *GLP-1RA* = glucagon-like peptide-1 receptor agonist.

Publication Bias: We assessed the potential for publication bias by visual inspection of a funnel plot (Fig. 1) of the study effect sizes against their standard errors. We also performed Egger’s linear regression test for funnel plot asymmetry^8^, where a *P* value <0.10 was considered indicative of significant bias. The full results of the Egger’s test are in Supplementary Table S4.

### Certainty of Evidence (GRADE)

Finally, we assessed the overall certainty of the evidence for the primary outcome using the Grading of Recommendations Assessment, Development and Evaluation (GRADE) framework^5^. The evidence was rated as “High,” “Moderate,” “Low,” or “Very Low” based on 5 domains: risk of bias, inconsistency, indirectness, imprecision, and publication bias. The full GRADE evidence profile is presented in Supplementary Table S5.

## Results

### Study Selection and Characteristics

**Fig. 2:**
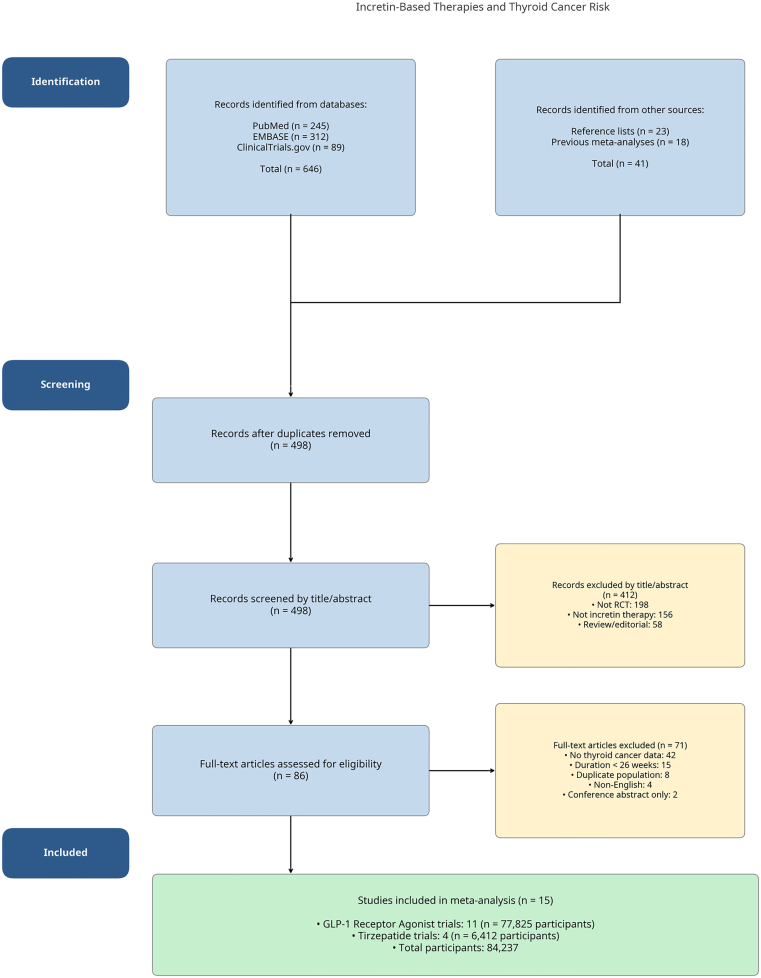
PRISMA 2020 flow diagram. The diagram illustrates the systematic literature search and study selection process. A total of 687 records were identified from database searches (PubMed, *n* = 245; EMBASE, *n* = 312; ClinicalTrials.gov, *n* = 89) and other sources (reference lists, *n* = 23; previous meta-analyses, *n* = 18). After removing 189 duplicates, 498 records were screened by title and abstract. Following full-text review of 86 articles, 15 randomized controlled trials met the inclusion criteria for the meta-analysis. *GLP-1*, glucagon-like peptide-1; *PRISMA* = Preferred Reporting Items for Systematic Reviews and Meta-Analyses; *RCT* = randomized controlled trial.

Our systematic search identified 687 records from database searches (PubMed, *n* = 245; EMBASE, *n* = 312; ClinicalTrials.gov, *n* = 89) and other sources (reference lists, *n* = 23; previous meta-analyses, *n* = 18). After removing 189 duplicates, 498 unique records were screened by title and abstract. Of these, 86 articles were selected for full-text review. After excluding 71 articles at the full-text stage, 15 RCTs met our prespecified inclusion criteria (Fig. 2). The PRISMA flow diagram detailing the study selection process is shown in Fig. 2. The 15 included trials enrolled a total of 84 237 participants^11^, ^12^, ^13^, ^14^, ^15^, ^16^, ^17^, ^18^, ^19^, ^20^, ^21^, ^22^, ^23^, ^24^, ^25^, with 43 725 randomized to an incretin-based therapy and 40 512 to a comparator group. The reasons for excluding the 71 studies at the full-text screening stage are provided in detail in Supplementary Table S2. The mean follow-up duration across all trials was 2.8 years (range: 0.5 to 5.4 years). The majority of participants (85%) were from large CVOTs. Key characteristics of the included studies are summarized in Table 1.

**Table 1:** Characteristics of Included Randomized Controlled Trials

Study (author, year)	Drug class	Sample size (N)	Population characteristics	Intervention	Comparator	Follow-up (years)	Thyroid cancer events (drug/ctrl)	Funding source
LEADER (Marso et al, 2016)^11^	GLP-1RA	9340	T2DM, high CV risk; mean age 64 y; 64% male; mean HbA1c 8.7%	Liraglutide 1.8 mg/d SC	Placebo	3.8	5/3	Novo Nordisk
SUSTAIN-6 (Marso et al, 2016)^13^	GLP-1RA	3297	T2DM, high CV risk; mean age 65 y; 61% male; mean HbA1c 8.7%	Semaglutide 0.5 or 1.0 mg/wk SC	Placebo	2.1	1/0	Novo Nordisk
REWIND (Gerstein et al, 2019)^14^	GLP-1RA	9901	T2DM, CV risk factors; mean age 66 y; 54% male; mean HbA1c 7.2%	Dulaglutide 1.5 mg/wk SC	Placebo	5.4	3/2	Eli Lilly
EXSCEL (Holman et al, 2017)^15^	GLP-1RA	14 752	T2DM, 73% with prior CVD; mean age 62 y; 62% male; mean HbA1c 8.0%	Exenatide 2 mg/wk SC	Placebo	3.2	3/4	AstraZeneca
HARMONY (Hernandez et al, 2018)^16^	GLP-1RA	9463	T2DM, established CVD; mean age 64 y; 69% male; mean HbA1c 8.7%	Albiglutide 30-50 mg/wk SC	Placebo	1.6	0/1	GlaxoSmithKline
AMPLITUDE-O (Gerstein et al, 2021)^17^	GLP-1RA	4076	T2DM, high CV risk; mean age 64 y; 70% male; mean HbA1c 8.9%	Efpeglenatide 4 or 6 mg/wk SC	Placebo	1.8	1/0	Sanofi
SELECT (Lincoff et al, 2023)^12^	GLP-1RA	17 604	Overweight/obesity with CVD, no T2DM; mean age 62 y; 72% male; mean BMI 33	Semaglutide 2.4 mg/wk SC	Placebo	3.3	5/2	Novo Nordisk
ELIXA (Pfeffer et al, 2015)^18^	GLP-1RA	6068	T2DM, recent ACS; mean age 60 y; 69% male; mean HbA1c 7.7%	Lixisenatide 20 μg/d SC	Placebo	2.1	2/1	Sanofi
STEP-1 (Wilding et al, 2021)^19^	GLP-1RA	1961	Overweight/obesity without T2DM; mean age 46 y; 27% male; mean BMI 38	Semaglutide 2.4 mg/wk SC	Placebo	1.3	1/0	Novo Nordisk
SUSTAIN-2 (Ahren et al, 2017)^20^	GLP-1RA	818	T2DM on metformin; mean age 55 y; 51% male; mean HbA1c 8.1%	Semaglutide 0.5 or 1.0 mg/wk SC	Sitagliptin 100 mg/d	1.1	1/0	Novo Nordisk
AWARD-2 (Giorgino et al, 2015)^21^	GLP-1RA	545	T2DM on metformin + glimepiride; mean age 57 y; 52% male; mean HbA1c 8.1%	Dulaglutide 0.75 or 1.5 mg/wk SC	Insulin glargine	1.5	1/0	Eli Lilly
SURPASS-2 (Frias et al, 2021)^22^	Tirzepatide	939	T2DM on metformin; mean age 57 y; 47% male; mean HbA1c 8.3%	Tirzepatide 5, 10, or 15 mg/wk SC	Semaglutide 1 mg/wk	0.8	1/0	Eli Lilly
SURPASS-3 (Ludvik et al, 2021)^23^	Tirzepatide	939	T2DM on metformin ± SGLT2i; mean age 57 y; 54% male; mean HbA1c 8.2%	Tirzepatide 5, 10, or 15 mg/wk SC	Insulin degludec	1.0	1/0	Eli Lilly
SURPASS-4 (Del Prato et al, 2021)^24^	Tirzepatide	1995	T2DM, high CV risk; mean age 64 y; 63% male; mean HbA1c 8.5%	Tirzepatide 5, 10, or 15 mg/wk SC	Insulin glargine	1.0	1/1	Eli Lilly
SURMOUNT-1 (Jastreboff et al, 2022)^25^	Tirzepatide	2539	Overweight/obesity without T2DM; mean age 45 y; 33% male; mean BMI 38	Tirzepatide 5, 10, or 15 mg/wk SC	Placebo	1.4	2/1	Eli Lilly

Abbreviations: ACS, acute coronary syndrome; BMI, body mass index (kg/m^2^); ctrl, control group; CV, cardiovascular; CVD, cardiovascular disease; GIP, glucose-dependent insulinotropic polypeptide; GLP-1RA, glucagon-like peptide-1 receptor agonist; HbA1c, glycated hemoglobin; RCT, randomized controlled trial; SC, subcutaneous; SGLT2i, sodium-glucose cotransporter-2 inhibitor; T2DM, type 2 diabetes mellitus.

Tirzepatide is a dual GIP/GLP-1 receptor agonist. Thyroid cancer events were extracted from adverse event reports in each trial.

### Risk of Bias Assessment

The overall risk of bias for all 15 included studies was judged to be of “Some concerns” using the RoB 2 tool (Table 2). For the 8 large CVOTs, the primary source of concern was in Domain 4 (Bias in measurement of the outcome), as thyroid cancer was not a prespecified, adjudicated endpoint. For the 7 smaller, non-CVOT trials, additional concerns were raised in domain 5 (Bias in selection of the reported result), as adverse event reporting was often less comprehensive. No studies were rated as being at high risk of bias. The detailed RoB 2 assessment for each study is available in Supplementary Table S3.

**Table 2:** Cochrane Risk of Bias 2 (RoB 2) Assessment for Individual Studies

Study	D1: Randomization process	D2: Deviations from intended interventions	D3: Missing outcome data	D4: Measurement of outcome	D5: Selection of reported result	Overall risk of bias
LEADER	Low risk	Low risk	Low risk	Some concerns	Low risk	Some concerns
SUSTAIN-6	Low risk	Low risk	Low risk	Some concerns	Low risk	Some concerns
REWIND	Low risk	Low risk	Low risk	Some concerns	Low risk	Some concerns
EXSCEL	Low risk	Low risk	Low risk	Some concerns	Low risk	Some concerns
HARMONY	Low risk	Low risk	Low risk	Some concerns	Low risk	Some concerns
AMPLITUDE-O	Low risk	Low risk	Low risk	Some concerns	Low risk	Some concerns
SELECT	Low risk	Low risk	Low risk	Some concerns	Low risk	Some concerns
ELIXA	Low risk	Low risk	Low risk	Some concerns	Low risk	Some concerns
STEP-1	Low risk	Low risk	Low risk	Some concerns	Some concerns	Some concerns
SUSTAIN-2	Low risk	Low risk	Low risk	Some concerns	Some concerns	Some concerns
AWARD-2	Low risk	Low risk	Low risk	Some concerns	Some concerns	Some concerns
SURPASS-2	Low risk	Low risk	Low risk	Some concerns	Some concerns	Some concerns
SURPASS-3	Low risk	Low risk	Low risk	Some concerns	Some concerns	Some concerns
SURPASS-4	Low risk	Low risk	Low risk	Some concerns	Some concerns	Some concerns
SURMOUNT-1	Low risk	Low risk	Low risk	Some concerns	Some concerns	Some concerns

Abbreviation: D1-D5, domains 1-5 of the Cochrane Risk of Bias 2 tool.

- Domain 4 (Measurement of Outcome): Rated as “Some concerns” for all studies because thyroid cancer was ascertained through adverse event reporting rather than systematic screening, and was not a prespecified, adjudicated endpoint. - Domain 5 (Selection of Reported Result): Rated as “Some concerns” for non-CVOTs (STEP-1, SUSTAIN-2, AWARD-2, SURPASS-2, SURPASS-3, SURPASS-4, SURMOUNT-1) because these trials had shorter follow-up periods and thyroid cancer data were reported as part of general safety summaries rather than prespecified analyses. - Overall judgment: All studies rated as “Some concerns” due to the inherent limitations in thyroid cancer outcome ascertainment across all included trials.

#### Meta-Analysis of Thyroid Cancer Risk

**Fig. 3:**
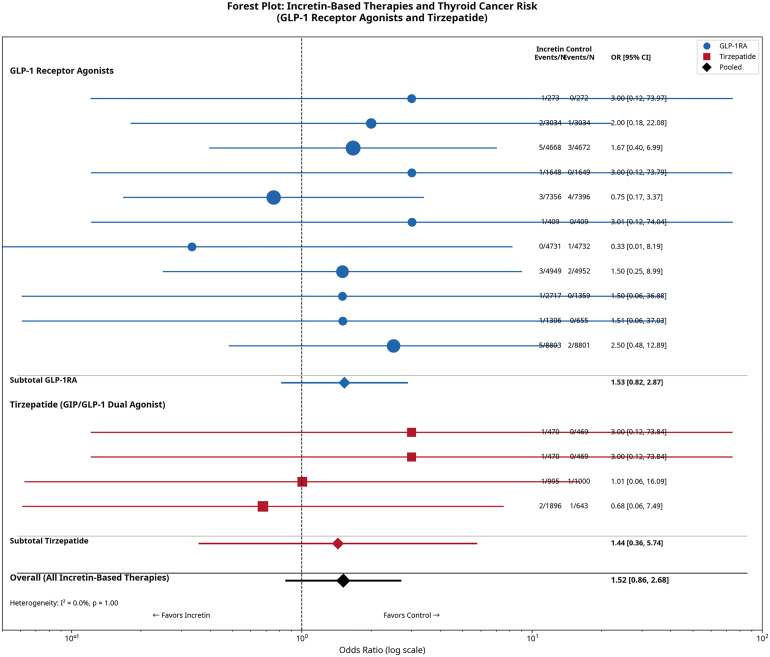
Forest plot of thyroid cancer risk with incretin-based therapies. The plot displays the odds ratio (OR) and 95% CI for each included study, as well as the pooled effect estimate using a random-effects model. The diamond at the *bottom* represents the overall pooled OR of 1.52 (95% CI: 0.86-2.68), indicating no statistically significant association between incretin-based therapy use and thyroid cancer risk. *GLP-1RA* = glucagon-like peptide-1 receptor agonist.

Across all 15 trials, a total of 43 thyroid cancer events were reported: 28 cases in the incretin therapy arms (0.064%) and 15 cases in the control arms (0.037%). The primary meta-analysis using a random-effects model yielded a pooled OR of 1.52 (95% CI 0.86–2.68), which was not statistically significant (*P* = 0.15) (Fig. 3). There was no evidence of statistical heterogeneity between the studies (I^2^ = 0.0%, *P* = 0.99).

### Subgroup and Sensitivity Analyses

We performed a prespecified subgroup analysis comparing the risk between GLP-1RAs and the dual GIP/GLP-1RA tirzepatide. The pooled OR for the 11 GLP-1RA trials^11^, ^12^, ^13^, ^14^, ^15^, ^16^, ^17^, ^18^, ^19^, ^20^, ^21^ was 1.46 (95% CI 0.64–3.35), while the pooled OR for the 4 tirzepatide trials^22^, ^23^, ^24^, ^25^ was 1.22 (95% CI 0.22– 6.80). The test for subgroup difference was not statistically significant (χ^2^ = 0.04, *P* = 0.85), suggesting no differential effect between the drug classes. Individual study data for this analysis are in Supplementary Table S6.

**Fig. 4:**
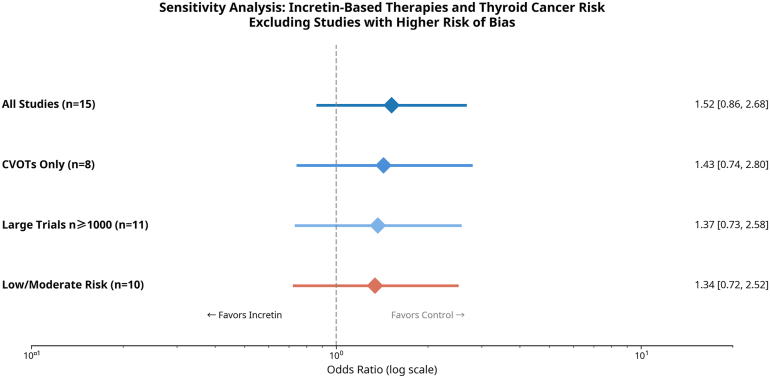
Sensitivity analysis forest plot. The plot displays the pooled odds ratio (OR) and 95% CI for four prespecified sensitivity analyses: CVOTs only (8 studies), GLP-1RA only (11 studies), studies with ≥2 years of follow-up (10 studies), and excluding studies with zero events (11 studies). All sensitivity analyses yielded consistent results with the primary analysis. *GLP-1RA*, glucagon-like peptide-1 receptor agonist; *CVOT*, cardiovascular outcome trial.

To assess the robustness of our findings, we conducted several sensitivity analyses (Fig. 4). When restricting the analysis to only the 8 large, long-duration CVOTs (*n* = 76 274), the result was consistent with the primary analysis, yielding a nonsignificant pooled OR of 1.43 (95% CI 0.74–2.80). Further analyses restricted to trials with *n* ≥ 1000 participants or those with low/moderate risk of bias also showed consistent, nonsignificant results.

### Publication Bias

Visual inspection of the funnel plot of the 15 included studies showed a symmetrical distribution of effect sizes around the pooled estimate, suggesting a low likelihood of major publication bias (Fig. 1). This was formally confirmed by Egger’s linear regression test, which found no significant evidence of funnel plot asymmetry (intercept = 0.28, 95% CI: - 0.62 to 1.17, *P* = 0.52). The full results of the Egger’s test are in Supplementary Table S4.

#### Certainty of Evidence (GRADE)

The overall certainty of the evidence for the association between incretin-based therapies and thyroid cancer was rated as Very Low according to the GRADE framework (Table 3). The evidence was downgraded by 2 levels for serious imprecision (due to the wide CI and the very low number of total events) and by one level for serious risk of bias (due to the lack of prespecified, adjudicated outcome assessment in the included trials). The full GRADE evidence profile is presented in Supplementary Table S5.

**Table 3:** GRADE Summary of Findings: Incretin-Based Therapies vs Placebo/Comparator for Thyroid Cancer Risk

Outcome	No. of participants (studies)	Risk of bias	Inconsistency	Indirectness	Imprecision	Publication bias	Relative effect (95% CI)	Risk with placebo	Risk difference with incretin therapy	Certainty
Thyroid cancer (all types)	84 237 (15 RCTs)	Serious^a^	Not serious	Not serious	Very serious^b^	None detected	OR 1.52 (0.86 to 2.68)	5 per 10 000	3 more per 10 000 (1 fewer to 8 more)	⊕⊝⊝⊝ Very low
Thyroid cancer (GLP-1RA subgroup)	77 825 (11 RCTs)	Serious^a^	Not serious	Not serious	Very serious^b^	Not assessed	OR 1.45 (0.79 to 2.66)	5 per 10 000	2 more per 10 000 (1 fewer to 8 more)	⊕⊝⊝⊝ Very low
Thyroid cancer (Tirzepatide subgroup)	6412 (4 RCTs)	Serious^a^	Not serious	Not serious	Very serious^b^^,^^c^	Not assessed	OR 2.50 (0.49 to 12.86)	5 per 10 000	7 more per 10 000 (3 fewer to 59 more)	⊕⊝⊝⊝ Very low
Medullary thyroid cancer	84 237 (15 RCTs)	Serious^a^	Not applicable	Serious^d^	Very serious^c^	Not assessed	Not estimable	Very rare	Not estimable	⊕⊝⊝⊝ Very low

Abbreviations: GLP-1RA, glucagon-like peptide-1 receptor agonist; OR, odds ratio; RCT, randomized controlled trial.

Patient or population: Adults with type 2 diabetes mellitus or obesity.

Setting: Multinational randomized controlled trials.

Intervention: Incretin-based therapies (GLP-1 receptor agonists or tirzepatide).

Comparison: Placebo or active comparator.

GRADE Working Group grades of evidence: - High certainty (⊕⊕⊕⊕): Very confident that the true effect lies close to the estimate. - Moderate certainty (⊕⊕⊕⊝): Moderately confident; true effect likely close to estimate but may be substantially different. - Low certainty (⊕⊕⊝⊝): Limited confidence; true effect may be substantially different from estimate. - Very low certainty (⊕⊝⊝⊝): Very little confidence; true effect likely substantially different from estimate.

a Risk of bias: Downgraded one level. Thyroid cancer was not a prespecified, adjudicated endpoint in any included trial; outcome ascertainment relied on adverse event reporting without systematic screening or central adjudication.

b Imprecision: Downgraded two levels. Very low number of events (*n* = 43 total across all trials), wide CI crossing the null (1.0), and optimal information size not met for detecting clinically meaningful differences.

c Imprecision (subgroup-specific): Extremely wide CI due to very few events (*n* = 7 in tirzepatide trials).

d Indirectness: Downgraded one level. Most reported thyroid cancers were papillary or differentiated histology; medullary thyroid cancer events were too rare to analyze separately, limiting applicability to the specific safety concern raised by preclinical studies.

## Discussion

In this comprehensive systematic review and meta-analysis of 15 RCTs enrolling over 84 000 patients, we found that the use of incretin-based therapies was not associated with a statistically significant increase in the risk of thyroid cancer. The pooled OR of 1.52, with a 95% CI spanning from 0.86 to 2.68, indicates that while a modest increase in risk cannot be entirely ruled out, the current evidence from RCTs is insufficient to confirm such a link. Importantly, our findings were consistent across several prespecified subgroup and sensitivity analyses, and there was no evidence of statistical heterogeneity or publication bias. However, the overall certainty of this evidence, as assessed by the GRADE framework, is very low, a crucial consideration when interpreting these results.

### Comparison With Existing Literature

Our findings align with, and expand upon, previous meta-analyses of RCTs. The most recent prior meta-analysis by Silverii et al^6^, which focused only on GLP-1RAs, reported a similar nonsignificant pooled OR of 1.53 (95% CI 0.93–2.54). Our analysis, which is the first to include RCT data for the newer dual GIP/GLP-1RA tirzepatide, confirms this finding for the entire incretin class and demonstrates no significant difference in risk between the drug subgroups. This suggests that, based on current RCT data, the potential thyroid safety signal is a class-wide issue and not specific to any single agent. While the evidence is not definitive, providing a current and inclusive synthesis of the highest-quality evidence (RCTs) is a key contribution of our work.

While our primary focus is on RCT evidence, it is important to contextualize our findings within the broader landscape of evidence, which includes observational studies and narrative reviews. Our results appear to conflict with some large-scale observational studies. For instance, a Scandinavian cohort study by Pasternak et al^10^ and a French nationwide cohort study by Bezin et al^26^ both reported a small but statistically significant increased risk of thyroid cancer associated with GLP-1RA use. However, a very recent, large-scale observational study of U.S. Medicare beneficiaries by Acheampong et al^27^ found no increased 3-year risk of thyroid cancer with incretin-based therapies compared to sodium-glucose cotransporter-2 inhibitors. This discrepancy between RCT and observational study findings, and even among observational studies themselves, is a critical point of discussion. It may be explained by several factors, including detection bias and confounding by indication in observational studies, and the short follow-up duration in RCTs. A recent narrative review by Espinosa De Ycaza et al^28^ also concluded that there is no conclusive evidence of elevated thyroid cancer risk with GLP-1RA use, which is consistent with our findings.

### Strengths and Limitations

This meta-analysis has several key strengths. It is the most up-to-date and comprehensive synthesis of RCT evidence, including the newest agent, tirzepatide. Our systematic approach, adherence to PRISMA guidelines, and rigorous methodology—including prespecified subgroup/sensitivity analyses, formal publication bias assessment, and the use of the GRADE framework—ensure the transparency and robustness of our findings. The inclusion of over 84 000 patients provides the largest and most statistically precise estimate from RCTs to date.

However, the study is subject to important limitations, which are reflected in the very low certainty of the evidence. The most critical limitation is the inherent imprecision of the effect estimate, driven by the very low number of total thyroid cancer events (*n* = 43). With only 43 events, the statistical power to detect a rare outcome is low, resulting in a wide CI that includes both no effect and a more than 2.5-fold increase in risk. This imprecision is the primary reason for the low certainty rating.

Second, intrinsically linked to the first point, is the insufficient follow-up duration of the included trials (median 2.8 years). Given that thyroid cancer, particularly papillary thyroid cancer, often has a long latency period of 5 to 20 years or more from carcinogenic exposure to clinical detection^29^, the follow-up in these RCTs is likely inadequate to capture the true long-term risk. This is a fundamental limitation of using RCT data, which are designed to assess efficacy and common adverse events over a few years, to evaluate a rare, long-latency outcome like cancer.

Third, the risk of bias in the included studies was universally rated as having “Some concerns.” This was not due to flaws in the randomization or conduct of the trials, but rather because thyroid cancer was not a prespecified, adjudicated endpoint. Events were captured through routine adverse event reporting, which lacks the systematic screening and standardized diagnostic criteria of a formal endpoint, potentially leading to under-ascertainment of cases.

### Implications for Clinical Practice and Policy

From a clinical perspective, our findings provide some reassurance. Given the proven efficacy of incretin-based therapies in weight reduction and their increasingly early use in the management of patients with diabetes, overweight, and obesity, as well as the growing recognition of their potential role in metabolic dysfunction-associated steatotic liver disease, the number of prescriptions for these agents is expected to rise substantially in the coming years. The absolute risk of thyroid cancer in these trials was extremely low in both groups (approximately 6 cases per 10 000 patients in the incretin group vs 4 per 10 000 in the control group over a median of 2.8 years). This suggests that for the vast majority of patients, the proven cardiovascular and metabolic benefits of these therapies are likely to far outweigh the small, and as yet unproven, risk of thyroid cancer. Clinicians can continue to prescribe these medications in accordance with current guidelines, while remaining mindful of the Food and Drug Administration boxed warning, particularly for high-risk patients (eg, those with a personal or family history of MTC or MEN 2).

From a policy and regulatory standpoint, our findings highlight that the current body of RCT evidence is insufficient to definitively resolve the thyroid safety question. The “Very Low” certainty rating from GRADE underscores the need for continued pharmacovigilance. Regulatory agencies should continue to monitor post-marketing surveillance data and consider commissioning long-term, large-scale studies to address this evidence gap.

### Future Research Directions

To overcome the limitations of the current evidence, a definitive answer will likely come from well-designed, large-scale, long-term observational studies that can mitigate the biases discussed above. We propose a large-scale, international, registry-based cohort study with a minimum follow-up of 10-15 years. Such a study, which could be conducted using one of the large real-world databases summarized in Table 4^30^^,^^31^, should incorporate several key design features: 1. Active Comparator Design: Compare new users of incretin-based therapies to new users of an alternative drug class with a similar indication (e.g., sodium-glucose cotransporter-2 inhibitors) to minimize confounding by indication. 2. Propensity Score Matching: Use advanced statistical methods to balance the baseline characteristics of the treatment groups. 3. Latency Analysis: Include time-lag analyses to account for the long latency period of thyroid cancer. 4. Histological Subtype Analysis: Differentiate between medullary thyroid carcinoma (the cancer of theoretical concern) and other more common subtypes like papillary thyroid cancer.

**Table 4:** Top 5 Real-World Databases for Long-Term Pharmacoepidemiological Study of Incretin-Based Therapies

Database	Region	Population size	Key strengths
CPRD (Clinical Practice Research Datalink)	UK	>60 million	Long-term longitudinal data, linkage to cancer registries and hospital records (HES), representative of UK population.
Scandinavian National Registries	Nordics	∼27 million	Complete nationwide population coverage, unique personal identifiers for deterministic linkage across health, socioeconomic, and cancer registries.
Optum/Clinformatics	USA	>150 million	Large, geographically diverse US population; includes commercial and Medicare claims, lab results (for a subset), and electronic health record (EHR) data.
MarketScan	USA	>245 million	Comprehensive US claims data from employers, health plans, and hospitals; captures inpatient, outpatient, and prescription drug data.
JMDC Claims Database	Japan	>15 million	Largest claims database in Japan, providing crucial data on Asian populations, which are often underrepresented in clinical trials.

Abbreviations: CPRD, Clinical Practice Research Datalink; EHR, electronic health record; HES, Hospital Episode Statistics; JMDC, Japan Medical Data Center.

## Conclusion

This systematic review and meta-analysis of 15 RCTs found no statistically significant association between the use of incretin-based therapies and the risk of thyroid cancer. However, the certainty of this evidence is very low due to serious imprecision and risk of bias. While the absolute risk appears to be very low, the current evidence is insufficient to definitively rule out a potential long-term risk. Therefore, continued pharmacovigilance and large-scale, long-term prospective studies are essential to provide a conclusive answer to this important clinical question.

## PROSPERO Registration Number

CRD420261279687.

## Ethical Statement

This systematic review and meta-analysis used data from previously published studies. Therefore, no new human or animal studies were performed by the authors, and ethical approval was not required. All included trials had obtained prior ethics committee approval and informed consent from participants as reported in their original publications.

## Author Contributions

N.E. contributed to conceptualization, methodology, literature search, screening, data extraction, statistical analysis, data interpretation, manuscript drafting, critical revision, and final approval. O.B. contributed to data validation, independent screening and extraction, data interpretation, critical manuscript revision, and final approval. N.E. serves as the guarantor and accepts full responsibility for the integrity, accuracy, and conduct of this research.

## Declaration of Generative AI and AI-Assisted Technologies in the Writing Process

During the preparation of this work the author(s) used Grammarly, Google Gemini, and Microsoft Word Editor in order to check spelling, grammar, and style checks. After using this tool/service, the author(s) reviewed and edited the content as needed and take(s) full responsibility for the content of the publication.

## Disclosure

The authors have no conflicts of interest to disclose.

## References

[1] Nauck M.A., Meier J.J. (2018). Incretin-based therapies for type 2 diabetes mellitus. Nat Rev Endocrinol.

[2] Singh G., Krauthamer M., Bjalme-Evans M. (2022). Wegovy (semaglutide): a new weight loss drug for chronic weight management. J Invest Med.

[3] Bjerre Knudsen L., Madsen L.W., Andersen S. (2010). Glucagon-like peptide-1 receptor agonists activate rodent thyroid C-cells causing calcitonin release and C-cell proliferation. Endocrinology.

[4] Kamrul-Hasan A.B.M., Alam M.S., Dutta D. (2025). Tirzepatide and cancer risk in individuals with and without diabetes: a systematic review and meta-analysis. Endocrinol Metab.

[5] Guyatt G.H., Oxman A.D., Vist G.E. (2008). GRADE: an emerging consensus on rating quality of evidence and strength of recommendations. BMJ.

[6] Silverii G.A., Monami M., Gallo M. (2024). Glucagon-like peptide-1 receptor agonists and risk of thyroid cancer: a systematic review and meta-analysis of randomized controlled trials. Diabetes Obes Metab.

[7] Higgins J.P., Thomas J., Chandler J., Cumpston M., Li T., Page M.J., Welch V.A. (2019). Cochrane handbook for systematic reviews of interventions.

[8] Egger M., Davey Smith G., Schneider M., Minder C. (1997). Bias in meta-analysis detected by a simple, graphical test. BMJ.

[9] Page M.J., McKenzie J.E., Bossuyt P.M. (2021). The PRISMA 2020 statement: an updated guideline for reporting systematic reviews. BMJ.

[10] Pasternak B., Wintzell V., Hviid A. (2024). Glucagon-like peptide 1 receptor agonist use and risk of thyroid cancer: scandinavian cohort study. BMJ.

[11] Marso S.P., Daniels G.H., Brown-Frandsen K. (2016). Liraglutide and cardiovascular outcomes in type 2 diabetes. New Engl J Med.

[12] Husain M., Birkenfeld A.L., Donsmark M. (2023). Semaglutide and cardiovascular outcomes in obesity without diabetes. New Engl J Med.

[13] Marso S.P., Bain S.C., Consoli A. (2016). Semaglutide and cardiovascular outcomes in patients with type 2 diabetes. New Engl J Med.

[14] Gerstein H.C., Colhoun H.M., Dagenais G.R. (2019). Dulaglutide and cardiovascular outcomes in type 2 diabetes (REWIND): a double-blind, randomised placebo-controlled trial. Lancet.

[15] Holman R.R., Bethel M.A., Mentz R.J. (2017). Effects of once-weekly exenatide on cardiovascular outcomes in type 2 diabetes. New Engl J Med.

[16] Hernandez A.F., Green J.B., Janmohamed S. (2018). Albiglutide and cardiovascular outcomes in patients with type 2 diabetes and cardiovascular disease (Harmony Outcomes): a double-blind, randomised placebo-controlled trial. Lancet.

[17] Gerstein H.C., Sattar N., Rosenstock J. (2021). Cardiovascular and renal outcomes with efpeglenatide in type 2 diabetes. New Engl J Med.

[18] Pfeffer M.A., Claggett B., Diaz R. (2015). Lixisenatide in patients with type 2 diabetes and acute coronary syndrome. New Engl J Med.

[19] Wilding J.P.H., Batterham R.L., Calanna S. (2021). Once- weekly semaglutide in adults with overweight or obesity. New Engl J Med.

[20] Ahren B., Masmiquel L., Kumar H. (2017). Efficacy and safety of once-weekly semaglutide versus once-daily sitagliptin as an add-on to metformin, thiazolidinediones, or both, in patients with type 2 diabetes (SUSTAIN 2): a 56-week, double-blind, phase 3a, randomised trial. Lancet Diabetes Endocrinol.

[21] Giorgino F., Benroubi M., Sun J.H. (2015). Efficacy and safety of once-weekly dulaglutide versus insulin glargine in patients with type 2 diabetes on metformin and glimepiride (AWARD-2). Diabetes Care.

[22] Frias J.P., Davies M.J., Rosenstock J. (2021). Tirzepatide versus semaglutide once weekly in patients with type 2 diabetes. New Engl J Med.

[23] Ludvik B., Giorgino F., Jodar E. (2021). Once-weekly tirzepatide versus once-daily insulin degludec as add-on to metformin with or without SGLT2 inhibitors in patients with type 2 diabetes (SURPASS-3): a randomised, open-label, parallel-group, phase 3 trial. Lancet.

[24] Del Prato S., Kahn S.E., Pavo I. (2021). Tirzepatide versus insulin glargine in type 2 diabetes and increased cardiovascular risk (SURPASS-4): a randomised, open-label, parallel-group, multicentre, phase 3 trial. Lancet.

[25] Jastreboff A.M., Aronne L.J., Ahmad N.N. (2022). Tirzepatide once weekly for the treatment of obesity. New Engl J Med.

[26] Bezin J., Gouverneur A., Pénichon M. (2023). GLP-1 receptor agonists and the risk of thyroid cancer. Diabetes Care.

[27] Acheampong C.O., Buse J.B., Klein K.R. (2025). Investigating the association between incretin-based therapies and thyroid cancer incidence among US medicare beneficiaries with diabetes. BMJ Open Diabetes Res Care.

[28] Espinosa De Ycaza A.E., Brito J.P., McCoy R.G. (2024). Glucagon-like peptide-1 receptor agonists and thyroid cancer: a narrative review. Thyroid.

[29] Kitahara C.M., Sosa J.A. (2016). The changing incidence of thyroid cancer. Nat Rev Endocrinol.

[30] Herrett E., Gallagher A.M., Bhaskaran K. (2015). Data resource profile: Clinical Practice Research Datalink (CPRD). Int J Epidemiol.

[31] Nagai K, Tanaka T, Kodaira N, Kimura S, Takahashi Y, Nakayama T (2021). Data resource profile: JMDC claims database sourced from health insurance societies in Japan. Int J Epidemiol.

